# No evidence of DUI in the Mediterranean alien species *Brachidontes pharaonis* (P. Fisher, 1870) despite mitochondrial heteroplasmy

**DOI:** 10.1038/s41598-022-12606-6

**Published:** 2022-05-20

**Authors:** Marek Lubośny, Beata Śmietanka, Marco Arculeo, Artur Burzyński

**Affiliations:** 1 Department of Genetics and Marine Biotechnology, Institute of Oceanology Polish Academy of Sciences, Sopot, Poland; 2 Department of Biological, Chemical and Pharmaceutical Sciences and Technologies, University of Palermo, Palermo, Italy

**Keywords:** Classification and taxonomy, DNA recombination, Development, Evolutionary biology, Sequencing, DNA sequencing, Next-generation sequencing, Sequence annotation, Reverse transcription polymerase chain reaction

## Abstract

Two genetically different mitochondrial haplogroups of *Brachidontes pharaonis* (p-distance 6.8%) have been identified in the Mediterranean Sea. This hinted at a possible presence of doubly uniparental inheritance in this species. To ascertain this possibility, we sequenced two complete mitogenomes of *Brachidontes pharaonis* mussels and performed a qPCR analysis to measure the relative mitogenome copy numbers of both mtDNAs. Despite the presence of two very similar regions composed entirely of repetitive sequences in the two haplogroups, no recombination between mitogenomes was detected. In heteroplasmic individuals, both mitogenomes were present in the generative tissues of both sexes, which argues against the presence of doubly uniparental inheritance in this species.

## Introduction

*Brachidontes pharaonis* (P. Fischer, 1870) is a Lessepsian mussel species that invaded the Mediterranean Sea after the opening of the Suez Canal in 1869 connecting the Indian Ocean through the Read Sea^1,2^. Due to intrinsic plasticity and overlapping morphological traits, this species is often mistaken for *B. variabilis*, which inhabits regions of the Indian Ocean and the West Pacific Ocean^3^. It is a gonochoristic species, with a white gonad-bearing mantle in males and brown mantle in females^4^. There is only one reported case of a clearly hermaphroditic individual^5^ in this species, and the sex determination system is otherwise quite stable.

Many bivalve species possess a unique system of mitochondrial inheritance called doubly uniparental inheritance (DUI)^6–8^. Under DUI male individuals are heteroplasmic with an additional, divergent mitogenome located mostly in their gonads. Furthermore, this divergent mitogenome is passed through the sperm to the progeny, unlike the male mitogenome of non-DUI animals, which is lost upon fertilization. In normal circumstances, germlines are homoplasmic towards one of the mitogenomes, M-type in males and F-type in females. However, in rare cases this second male-type mitogenome can also be detected in female individuals (for such exceptions, see^9–11^). After fertilization, initially heteroplasmic embryos behave in one of two ways. If the embryo is a male, the M-type mitochondria group together during the first cell divisions, becoming the dominant mitochondrial fraction in gonads of adult male (somatic tissues are mostly dominated by F-type mitochondria). On the other hand, if the embryo develops into a female individual, the M-type mitochondria get dispersed during the first few division cycles and the signal from the M-type mitochondrial DNA disappears. The mechanism of this elimination is unknown^12–22^.

Genetic studies based on *cox1* and 16S rRNA gene markers revealed the presence of two (p-distance 6.8%) different haplogroups (M and L; referred to respectively A and B later on due to possible misinterpretation of the M haplogroup as male-type mtDNA) in *B. pharaonis,* suggesting the existence of cryptic species. No heteroplasmic individuals were identified within the Mediterranean population, and the presence of a particular mitochondrial haplogroup did not correlate with the sex of the individuals^1,3,23^. Nevertheless, many studies suggested the presence of DUI in this genus^3,16,19,24–26^. However, there are well-known difficulties with detecting DUI by end-point PCR with universal primers^27^. The variability of the sequence divergence between mitogenome pairs ranges from around 5% to over 50%^16,28,29^. This makes the whole approach problematic for the following reasons. In the cases of low divergence, the amplification of the most prevalent mitogenome, or the one with sequence slightly better matching with PCR primers, may mask the presence of the second mitogenome. In the cases of high divergence, universal primers may not be universal enough to pick up the second genome at all. Moreover, regardless of the divergence, there is always a possibility that a mitogenome of another, contaminating, biological entity, would be co-amplified. This is facilitated by high sensitivity of end-point PCR protocols^12,21,27^. All this prompted us to use a quantitative mitogenomic approach to further corroborate the possibility of DUI in this species.

## Results

**Figure 1:**
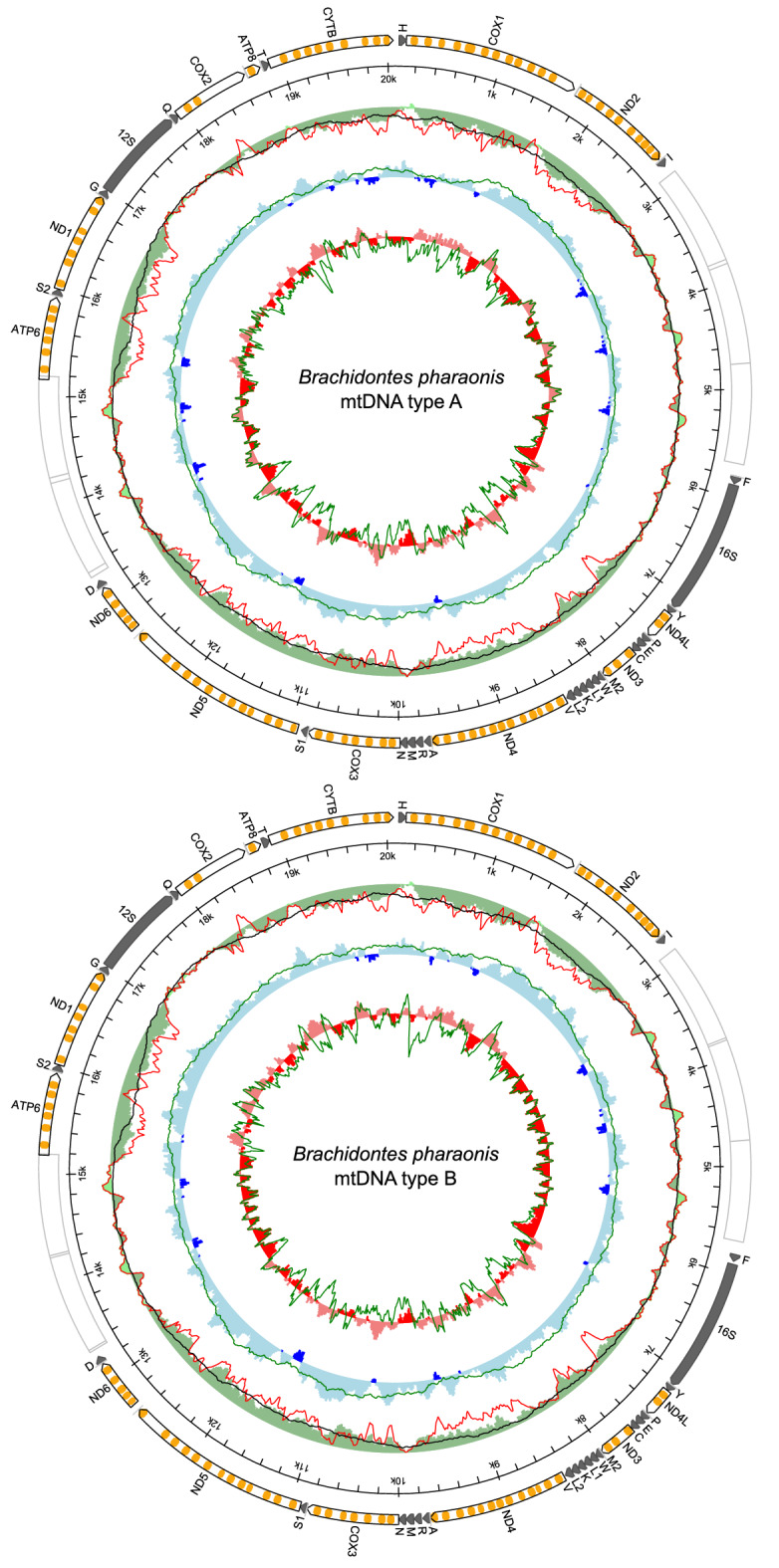
Genetic map of *Brachidontes pharaonis* mitochondrial genomes. The white arrows with orange bands represent protein-coding genes with predicted transmembrane domains. The dark arrows represent the rRNA and tRNA genes; the white boxes indicate the location of repetitive sequences. The figure and compositional indices were generated with MITOCONSTRICTOR as in^33,34^.

We were able to detect and sequence two slightly divergent mitogenomes in *Brachidontes pharaonis* samples (p-distance 0.071 for coding genes and 0.086 for the whole mtDNA). The preliminary results of the end-point PCR (Table 1) suggested not only the existence of heteroplasmy, but also showed that it was not linked with sex of individuals in this species. The results between PCR and qPCR differ because not every homoplasmic individual in PCR was checked with qPCR and some of the heteroplasmic individuals were resolved as homoplasmic after the qPCR. This was due to the very low estimated copy number of the second mitogenome, much lower than the copy number of a nuclear gene. Very high Cq values, close to the NTC control, were observed in these samples. Since such results could be either caused by non-specific signal (primer-dimer formation) or the truly very low gene copy number (much lower than that of atpα nuclear gene), these were classified as likely caused by sample contamination and not by true heteroplasmy. In the absence of any sex-bias, instead of naming those mitogenomes as M-type for male and F-type for female, we will refer to them neutrally as type A and type B. The mitogenomes (Fig. 1) are similar in length (type A: 20,066 bp, type B: 20,072 bp), code the typical set of 13 proteins, 2 rRNAs and 23 tRNAs, all on the same strand and in the same order. The striking feature of both mitogenomes is the presence of two long noncoding regions, rich in repetitive sequences, that divide the mitogenomes almost perfectly in half. Fragments of open reading frames in noncoding regions were identified as repeats of the 5′ end of *atp6*, suggesting the occurrence of multiple tandem duplication random loss events that encompass fragments of the coding sequences, throughout the evolution of these mitogenomes.

**Table 1:** Detection of the two mitogenomes (A and B) by PCR and qPCR.

PCR	All	Female	Male	qPCR	All	Female	Male
Only A	11	7	4	Only A	8	3	5
Only B	15	8	7	Only B	15	6	9
A and B	13	6	7	A and B	7	4	3
N	39	21	18	N	30	13	17
				A > B	3	1	2
				B > A	4	3	1

Number of tested individuals (N) in which A or B mitogenome was detected. For qPCR, each heteroplasmic individual showed an excess of one mitogenome over the other, which is also indicated here for mantle tissues.

**Figure 2:**
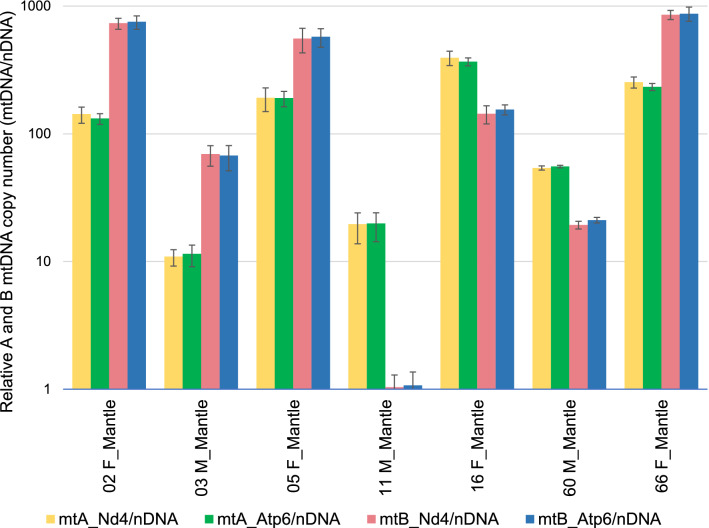
The ratio of mtDNA to nDNA in seven heteroplasmic individuals of *B. pharaonis.* F represents female individuals; M represents male individuals. The yellow and green colored bars represent sequence copy number per nuclear DNA for type A mtDNA; The pink and blue bars represent sequence copy number per nuclear DNA for type B mtDNA. The target genes *atp6* and *nd4* are located on the opposite sites of the respective mitogenomes separated by repetitive sequences. Samples described as “Mantle” contained mainly gonadal tissues, not just the somatic mantle.

Given the overall relatively small divergence in mitochondrial sequences, the long repetitive sequences, and heteroplasmy, the possibility of widespread mitochondrial recombination at one of the long noncoding regions was considered. Such recombination would impair our ability to detect the type of mitogenome with just one small qPCR fragment. To address this issue, four pairs of specific qPCR primers were used to quantify the two parts of mitogenomes A and B separately (Supplementary data Table S1 and S2). Under the no recombination hypothesis the four quantities, one per part of the mitogenome, should match according to the mitogenome type, whereas if recombination is involved, the four estimates would be mismatched. Analysis of 30 *B. pharaonis* individuals revealed no noticeable difference between the halves of mitogenome copy number (Fig. 2). We have observed seven cases of heteroplasmy divided more or less evenly (no statistically significant differences for Fisher’s exact test) between sexes. The heteroplasmic individuals were: 4 females, 3 males in qPCR (23%) and 6 females, 7 males in end-point PCR analysis (33%). Both A and B mitogenomes were detectable in males and females (Supplementary data Table S5–S10 and Fig. S1).

**Figure 3:**
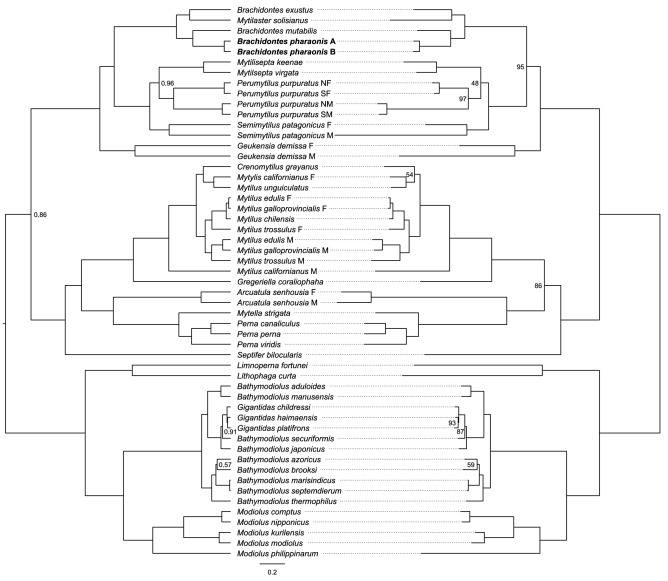
The phylogenetic position of *Brachidontes pharaonis* mitogenomes among mitogenomes of other mytilid mussels. The left tree represents Bayesian phylogenetic analysis done in BEAST2, and the right tree represents maximum likelihood analysis done in IQ-TREE. The nodes support values equaled 1 (Posterior probabilities, left) and 100% (bootstrap values, right), unless indicated differently.

The newly reported mitogenomes of *B. pharaonis* form a monophyletic clade within the *Brachidontes* genus clade (Fig. 3). *Brachidontes* is a large genus composed of around 50 individual species (WoRMS database). However, due to the small number of available complete mitogenomes, the analysis of taxonomic affinities of the mitogenomes, including possible mitochondrial introgression events within this genus can not be conclusive. As a side note, phylogenetic analysis revealed the following inconsistency: genus *Brachidontes* seems to be paraphyletic, due to the presence of *M. solisianus* mitogenome within the clade. This hints that the taxonomy of *M. solisianus* may require reconsideration. Nevertheless, the phylogenetic distance between the two *B. pharaonis* mitogenomes is similar to the intraspecies distance between F-type mitogenomes form north and south populations of the Chilean mussels *Perumytilus purpuratus* which were separated from each other in Pleistocene^30–32^. It is also greater than the distance between several interspecies pairs of mitogenomes: *Mytilus edulis* and *M. galloprovincialis*, *Bathmodiolus marisindicus* and *B. septemdierum,* as well as all available mitogenomes form genus *Gigantidas.*

## Discussion

We reported two mitogenomes (p-distance 8.6%) in *B. pharaonis*. These two mitogenomes are quite frequently present in the same individuals (Table 1). Is it possible that the observed heteroplasmy was not true but the result of a recent *numt* which is segregated in the population? We dismiss this possibility for the following reasons. Both mitogenomes were highly expressed at RNA level (31% of all NGS RNAseq sequencing reads mapped onto the A-type mtDNA and 4% onto the B-type mtDNA sequence), which is not a typical feature of a *numt*. Moreover, *numts* are usually much smaller than the complete mitogenomes (but look here^35^), and their sequence is degenerated^36^. Here both very large mitogenomes have all their genes intact and seemingly functional. Finally, the ratios of copy numbers of mitogenomes to the nuclear genome are not constant. A tight correlation between a *numt* and nuclear copy number is expected, which is not the case here. What can be the reason for the existence of two such haplotypes within this species?

Since DUI was postulated for some *Brachidontes* species based solely on sequence divergence, it is reasonable to consider it here^3,24^. One may ask what sequence divergence threshold (if there is one) can be unequivocally associated with DUI? The situation present in the Baltic Sea *Mytilus* mussels^37,38^, where the divergent paternal mitogenome was replaced by the masculinized F-type mitogenome, is exceptional. Consequently, besides the differences in the noncoding control region, the M and F mitogenomes are identical in that case. If such extreme cases are not considered, the lowest divergence of the complete mitochondrial genomes observed and counted as DUI belongs to *Arctica islandica*^28,29^, with the M-F divergence (nucleotide p-distance) at 5.1% for coding genes and 6.9% for the whole mtDNA (Fig. S2). Even if a wider species range is considered, for which only *cox1*^16^ gene fragments are known, the least divergent is also *Arctica islandica:* 2.5% at the protein level and 5.2% at the nucleotide level. So, can we count the divergence observed in *B. variabilis*^3^ 3.6% at the nucleotide level as a suggestion of DUI existence, with the lowest known divergence in the *cox1* gene? Or is this just a high haplotype diversity within a species, not linked to sex-related heteroplasmy? This rhetorical question also applies to *B. pharaonis*.

In the context of DUI, divergence alone is just a secondary factor, depending only on the time of separation of the two lineages and their evolutionary rates. Nevertheless, for the emerging mitogenomes to play different physiological roles, there must be functional differences between their proteins, which is problematic for low genetic distances. However, even with a small number of nonsynonymous substitutions in the sequence, the encoded protein may become different enough (like in quickly evolving viruses or targeted enzyme engineering^39,40^) to differ in activity. Unfortunately, tracing such substitutions in the context of DUI is difficult, but the possibility that a low-divergence mitogenome may still play distinct physiological role can’t be discounted. Are there any other features that could help classify a mitogenome as involved in DUI? Perhaps we should consider additional open reading frames (ORFs) or gene extensions with no homology to the second mitogenome as a DUI marker? Is this a universal feature of DUI mitogenomes? Indeed, this seems to be true for all known pairs of DUI mitogenomes (Table 2): each of them has some gender-specific structural features, but this is not the case for *B. pharaonis.* Here, both mitogenomes are structurally identical.

**Table 2:** Features correlating with doubly uniparental inheritance observed in complete pairs of mitogenomes (when both M and F are available).

Family	Species	Feature
Mytilidae	*Arcuatula senhousia*^41^	duplicated *m-cox2* with 3′ extension
	*Geukensia demissa*^33^	*m-cox2* extension
	*Mytilus californianus*^42^	f-ORF; a bit longer *m-atp8* gene
	*Mytilus galloprovincialis*^43^	f-ORF; a bit longer *m-atp8* gene
	*Mytilus edulis*^44^	f-ORF; a bit longer *m-atp8* gene
	*Mytilus trossulus*^45^	f-ORF; a bit longer *m-atp8* gene
	*Perumytilus purpuratus*^30^	*nd2*-like f-ORF
	*Semimytilus patagonicus*^34^	*m-atp8* extension; ORFs
Veneridae	*Ruditapes philippinarum*^46^	m-ORF *(rphm21)*
	*Meretrix lamarckii*^47^	Insertion in *m-cox2*; additional ORFs
Semelidae	*Scrobicularia plana*^48^	Insertion in *m-cox2*; additional m-ORFs
Tellinidae	*Limecola balthica*^48^	Insertion in *m-cox2*; additional m-ORFs
Arcticidae	*Arctica islandica*^28^	f-ORFs*: nd6* × *nd2* hybrid gene duplication
Hyriidae	*Echyridella menziesii*^15^	*m-cox2* 3′ extension; ORFs
Margaritiferidae	*Margaritifera margaritifera*^49^	*m-cox2* 3′ extension; ORFs
	*Pseudunio marocanus*^50^	*m-cox2* 3′ extension; ORFs
	*Cumberlandia monodonta*^15^	*m-cox2* 3′ extension; ORFs
Unionidae	*Venustaconcha ellipsiformis*^51^	*m-cox2* 3′ extension; ORFs
	*Utterbackia peninsularis*^52^	*m-cox2* 3′ extension; ORFs
	*Unio tumidus*^53^	*m-cox2* 3′ extension; ORFs
	*Unio pictorum*^54^	*m-cox2* 3′ extension; ORFs
	*Unio delphinus*^55^	*m-cox2* 3′ extension; ORFs
	*Unio crassus*^56^	*m-cox2* 3′ extension; ORFs
	*Solenaia carinata*^57^	*m-cox2* 3′ extension; ORFs
	*Quadrula quadrula*^51^	*m-cox2* 3′ extension; ORFs
	*Pyganodon grandis*^51^	*m-cox2* 3′ extension; ORFs
	*Potamilus alatus*^58^	*m-cox2* 3′ extension; ORFs
	*Lampsilis siliquoidea*^59^	*m-cox2* 3′ extension; ORFs
	*Lampsilis powellii*^59^	*m-cox2* 3′ extension; ORFs
	*Aculamprotula tortuosa*^60^	*m-cox2* 3′ extension; ORFs
	*Lamprotula leai*	*m-cox2* 3′ extension
	*Pronodularia japanensis*^51^	*m-cox2* 3′ extension; ORFs
	*Sinohyriopsis cumingii*^51^	*m-cox2* 3′ extension; ORFs
	*Anodonta anatina*^61^	*m-cox2* 3′ extension; ORFs
	*Sinanodonta woodiana*^62^	*m-cox2* 3′ extension; ORFs
	*Lanceolaria lanceolata*^63^	*m-cox2* 3′ extension; ORFs
	*Potomida littoralis*^64^	*m-cox2* 3′ extension; ORFs
	*Microcondylaea bonellii*^65^	*m-cox2* 3′ extension; ORFs
	*Chamberlainia hainesiana*^64^	*m-cox2* 3′ extension; ORFs
	*Pilsbryoconcha exilis*^64^	*m-cox2* 3′ extension; ORFs
	*Monodontina vondembuschiana*^64^	*m-cox2* 3′ extension; ORFs

Another feature of mtDNA, which has been reported in the context of DUI, is mitochondrial recombination^65,66^. The existence of very large repetitive elements suggests, that at least intramolecular recombination would be present in *B. pharaonis*. Yet, we have not found any evidence for the exchange of large parts between A and B mitogenomes. They apparently maintain their integrity, despite the conditions, which should favor the exchange^67^. The qPCR method would not detect low frequency, short span recombination so this has to be considered cautiously. However, no detectable signature of recombination is present within the A and B mitogenomic sequences, therefore even if such recombination is present, it is limited to somatic cells.

**Figure 4:**
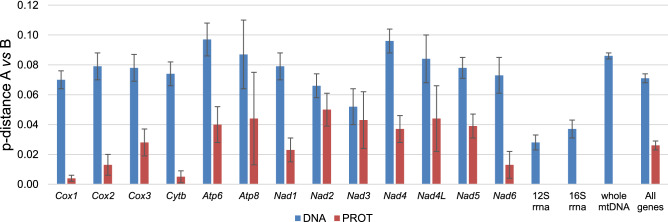
The divergence between A and B type mitogenome of *B. pharaonis.* The blue color represents the p-distance for nucleotide sequences, and dark red color represents p-distance for translated protein genes.

Several studies postulated the existence of cryptic species within the *Brachidontes* genus (*B. pharaonis/variabilis*^3^ and *B. puniceus/exustus*^24,68^). These were usually argued by the distinct divergence (p-distance) at the nucleotide level (~ 7–20%) between mitochondrial sequences. However, at the protein level, these distances were much lower (p-distance ~ 0–1.5%). These must be interpreted cautiously because only short fragments of one mitochondrial gene are available (*cox1:* AY621835-AY621860; AY621862-AY621865; AY621909; AY621911; AY825105-AY825108; DQ836012; DQ836013; DQ836019-DQ836021) and the mitogenomic distances may be quite different (Fig. 4). However, all nonsynonymous substitutions in the mentioned *cox1* fragments stay within the group of nonpolar, mostly aliphatic amino acids (Ile, Val, Leu, and in two cases also Met and Phe). This suggests no alternation in the overall protein structure/reactivity as is indicated by amino acid substitution matrices^69,70^ derived from empirical data. The issue of cryptic species identification becomes even more complicated when specimens are erroneously assigned^68^ to their respective species, as in the case of *Brachidontes/Mytilaster* genera. This is the example of *Mytilaster solisianus* (d'Orbigny, 1842)*,* known earlier as *Brachidontes solisianus* (d'Orbigny, 1842). The current taxonomy classifies this species as *Mytilaster*, but the mitogenomic phylogenetic tree (Fig. 3) does not support this. Furthermore, the *M. solisianus* mitogenome deposited in GenBank has been wrongly annotated as *Perna perna*^71^. A taxonomic revision of *Brachidontes* seems warranted but should not be based solely on mitochondrial markers, which are known to evolve under strong selective constraints and are prone to introgression^72,73^. A good example of potential pitfalls associated with such a simplistic use of mitochondrial markers comes from the case of Baltic *Mytilus trossulus,* which in fact is a nuclear hybrid of *M. edulis* and *M. trossulus* that lost their native mitochondrial genome towards one from *M. edulis*^72,74,75^ (also see here for M/F recombination^66,76^). If only the mitogenomic protein level p-distances are considered, the cryptic species hypothesis is becoming less likely. However, the fact that heteroplasmic individuals were consistently observed suggests that anomalies in the mitochondrial inheritance may be involved.

In conclusion, *B. pharaonis* represents a species possessing two slightly different mitogenomes (p-distance 8.6%) in every possible combination between the sexes. Total homoplasmy for mitogenomes A or B, as well as heteroplasmy of both mitogenomes within a single individual are possible. This heteroplasmy is not correlated with sex, which excludes DUI. Did we observe the first stages of emerging DUI in this species or is heteroplasmic *B. pharaonis* a hybrid of two very similar cryptic species and the observed heterpolasmy represents paternal leakage? Future studies concerning *Brachidontes* populations are needed to conclusively answer this question. On a more practical level, we advise that the use of somatic tissues^77^, in phylogenetic studies on bivalves, usually practiced as a precaution to avoid amplification of the potential M mitogenome, may not always work as planned^78^. In the case of *B. pharaonis* such an approach would lead to seemingly random amplification of one of the two mitogenomes present in an individual.

## Methods

Samples of *Brachidontes pharaonis* mussels were gathered in June 2014–2015 at the salt pan “infersa” of the Marsala lagoon (northwest of Sicily—Italy). Individuals were sectioned with a sterile scalpel blade, checked for male or female gametes under a light microscope, and stored frozen in − 80 °C until further use. DNA was extracted using the modified CTAB extraction method^79,80^. Small tissue samples (~ 50 mg) were incubated overnight in the 700 µl of extraction buffer (2% CTAB, 0.1 M Tris–HCl, 1.4 M NaCl, 20 mM EDTA, 1 mg/ml proteinase K and 35 mM 2-mercaptoethanol). The digested samples were then extracted with chloroform (1:1 vol/vol ratio) and centrifuged three times at 20,000 × g for 10 min. Then the DNA remaining in the aqueous phase was precipitated by mixing with cold isopropanol (1:1 vol/vol), incubated for 20 min at − 20 °C, and centrifuged (20,000 × g for 30 min at 4 °C). The recovered DNA pellets were washed twice with 75% ethyl alcohol and dried in a vacuum concentrator. At the final stage, DNA pellets were resuspended in Tris–EDTA buffer, checked for DNA concentration and integrity by gel electrophoresis and Epoch microplate Spectrophotometer. RNA was extracted with the GenElute Mammalian Total RNA miniprep kit (Sigma).

Total RNA from three mantles of male individuals was pooled and sent to Macrogen Inc (Korea) for high throughput NGS sequencing (MiSeq Illumina, TruSeq NGS library 2 × 150 bp). Raw sequencing reads have been submitted to the SRA GenBank database under accession number SRR19141670. The acquired data were processed according to the Oyster river protocol^34,81^ and assembled into the raw transcriptome. Long-range PCR primers for amplification of overlapping mitogenome fragments have been designed based on assembled contigs containing mitochondrial genes identified with Wise2 software^82^. PCRs were carried out in a volume of 20 µl, containing 25 ng of DNA, primers at 0.4 µM each, dNTP at 200 µM, 1.5 mM MgCl_2,_ and 0.4 U Phusion High-Fidelity polymerase (Thermo Scientific) suspended in GC buffer for difficult GC-rich templates. The PCR conditions were as follows: initial denaturation at 98 °C for 30 s; 35 cycles of denaturation at 98 °C for 10 s, annealing (Table S3 and S4) for 30 s and extension at 72 °C for 8 min. The final extension at 72 °C lasted 10 min. The amplified products were then assigned to their respective mitogenome (A and B) and sent for another NGS sequencing (MiSeq Illumina, TrueSeq NGS library, 2 × 300 bp). Complete mitochondrial DNA sequences have been recovered with NOVOplasty^83^ software and validated by mapping NGS reads onto the assembled mitogenomes in CLC Genomics Workbench 9.5 (QIAGEN). The two acquired mitogenomes were then annotated with CRITICA^84^, Wise2^82^, GLIMMER^85^, ARWEN^86^, and nhmmer^87^. Mitogenome circular diagrams and compositional indices were drawn with MITOCONSTRICTOR^33,34^ (https://github.com/aburzynski/mitoconstrictor). Annotated mitochondrial genomes for *Brachidontes pharaonis* were deposited in the GenBank under accession numbers ON464163 and ON464164.

Based on assembled transcriptome and two mitogenome sequences, a set of five qPCR primer pairs spanning: nuclear *atpα* gene and mitochondrial *nad4*, *atp6* (from both mitogenomes; type A and type B) were designed in Primer3^88^. The specificity of each primer pair was verified, and there was no cross-amplification between A and B mitogenomic fragments. Quantitative PCR efficiency has been verified by running standard curves in nine repetitions with seven dilution points of samples. Reactions quantifying A and B mtDNA, as well as nuclear DNA, were performed on an ECO48 (Illumina/now PCRmax) Real-Time PCR System according to the qPCR kit (EurX) manufacturer instructions, in 10 µl reaction volume containing 1 × SG qPCR Master Mix, 2 µl of DNA (at ~ 15 ng/µl) and 0.5 µM of each primer. The thermal profile was as follows: initial denaturation at 95 °C for 10 min, followed by 35 cycles of 10 s denaturation at 94 °C, annealing at 60 °C for 30 s, elongation at 72 °C for 30 s, and a melting curve 55–95 °C. Primer sequences, reaction efficiency, and tabulated results have been placed in Supplementary data S1 and S2. Statistical Fisher’s exact test (Statistica 7, StatSoft) was used to calculate the significance of the association between sex and heteroplasmy.

Reconstruction of phylogenetic relations within the Mytilidae family was based on 53 mitogenomes (Table 3). All mitogenomes from the Mytilidae family available GenBank database (accessed May 2021) were used. The 12 mitochondrial protein coding genes (*atp8* was omitted due to high divergence and in a few cases annotation problems^89^) were used. Each gene was aligned separately, at protein level in MEGA7^90^ software, with the ClustalW algorithm, to ensure that the codon structure of each gene is retained at the alignment level. Gap Extension and Gap Opening costs were set at 5, and the final alignments were visually inspected. No obvious alignment problems were encountered. Phylogenetic reconstruction was done using two approaches: Bayesian inference (BI) and Maximum Likelihool (ML). The same set of individual gene alignments (as separate data partitions) was used in both analyses.

**Table 3:** List of mitogenomes used in phylogenetic analysis, with species, accession numbers, and references (wherever available).

Species and references	Acc. no	Species and references	Acc. no
*Arcuatula senhousia F*^41^	GU001953	*Modiolus nipponicus*^94^	MK721547
*Arcuatula senhousia M*^41^	GU001954	*Modiolus philippinarum*^95^	KY705073
*Bathymodiolus aduloides*^96^	MT916741	*Mytella strigata*	MT991018
*Bathymodiolus azoricus*^96^	MT916742	*Mytilaster solisianus*^71^	KM655841
*Bathymodiolus brooksi*^96^	MT916743	*Mytilisepta keenae*^94^	MK721542
*Bathymodiolus japonicus*^97^	AP014560	*Mytilisepta virgata*^94^	MK721548
*Bathymodiolus marisindicus*^96^	MT916745	*Mytilus californianus F*^42^	GQ527172
*Bathymodiolus manusensis*	KY270856	*Mytilus californianus M*^42^	GQ527173
*Bathymodiolus securiformis*	NC_039552	*Mytilus chilensis*^98^	KT966847
*Bathymodiolus septemdierum*^97^	AP014562	*Mytilus unguiculatus*^99^	KJ577549
*Bathymodiolus thermophilus*^94^	MK721544	*Mytilus edulis F*^89^	MF407676
*Brachidontes exustus*^100^	KM233636	*Mytilus edulis M*^44^	HM489874
*Brachidontes mutabilis*^94^	MK721541	*Mytilus galloprovincialis M*^43^	FJ890850
*Brachidontes pharaonis A*	ON464163	*Mytilus galloprovinialis F*^43^	FJ890849
*Brachidontes pharaonis B*	ON464164	*Mytilus trossulus F*^45^	HM462080
*Crenomytilus grayanus*^94^	MK721543	*Mytilus trossulus M*^45^	HM462081
*Geukensia demissa F*^33^	MN449487	*Perna canaliculus*^101^	MG766134
*Geukensia demissa M*^33^	MN449488	*Perna perna*	MT588202
*Gigantidas childressi*^96^	MT916744	*Perna viridis*^102^	JQ970425
*Gigantidas haimaensis*^96^	MT916746	*Perumytilus purpuratus NF*^30^	MH330332
*Gigantidas platifrons*^97^	AP014561	*Perumytilus purpuratus NM*^30^	MH330330
*Gregeriella coraliophagia*^94^	MK721545	*Perumytilus purpuratus SF*^30^	MH330333
*Limnoperna fortunei*^103^	KP756905	*Perumytilus purpuratus SM*^30^	MH330331
*Leiosolenus lischkei*^94^	MK721546	*Semimytilus patagonicus F*^34^	MT026712
*Modiolus comptus*^96^	MN602036	*Semimytilus patagonicus M*^34^	MT026713
*Modiolus kurilensis*	KY242717	*Septifer bilocularis*^94^	MK721549
*Modiolus modiolus*^104^	KX821782		

All species names have been updated according to the WoRMS (World Register of Marine Species) database (accession date July 2021).

BI was performed in BEAST2^91^. The parameters were as follows: nucleotide substitution model GTR + I + 4G, based on the results from the bModelTest package for BEAST2 software, relaxed log-normal clock, due to the varying evolution rates between F and M-type mtDNA and Yule prior to the common tree. The Markov chain was run in four replicates for 10^7^ generations and sampled every 10,000th step. The convergence of samples was checked with Tracer^92^, the effective sample size for estimated parameters was greater than 200. ML was performed in IQ-TREE^93^, under the default parameters with ultrafast bootstrap approximation parameter set to 10,000 replicates. Substitution models for every partition were chosen with build-in ModelFinder tool. Model GTR + F + I + 4G was the best fitting for all protein genes except *nd2*, *nd4*, *nd5* for which GTR + F + R5 was chosen.

## Supplementary Information

Supplementary Information.
