# Supplementary material for: No evidence of DUI in the Mediterranean alien species Brachidontes pharaonis (P. Fisher, 1870) despite mitochondrial heteroplasmy
Source: Sci Rep. 2022 May 20;12:8569. doi: 10.1038/s41598-022-12606-6 (PMC9122905; doi:10.1038/s41598-022-12606-6)
Supplement: Supplementary file 1 — Supplementary Information. [file 41598_2022_12606_MOESM1_ESM.pdf]

Supplementary Information for:

No evidence of DUI in the Mediterranean alien species *Brachidontes pharaonis* (P. Fisher, 1870) despite mitochondrial heteroplasmy

Marek Lubośny[1*], Beata Śmietanka[1], Marco Arculeo[2], Artur Burzyński[1]

[1]Department of Genetics and Marine Biotechnology, Institute of Oceanology Polish Academy of Sciences, Sopot, Poland

[2] Department of Biological, Chemical and Pharmaceutical Sciences and Technologies, University of Palermo, Palermo, Italy

*Corresponding Author: Marek Lubośny (lubosny@iopan.pl)

Extended Data Table S1. qPCR Standard curves efficiency

Gene	Equation	Efficiency	R^2
ATPα nuclear DNA	y=-3.41x+35.31	96.58%	0.998
Nad4 type A mtDNA	y=-3.45x+ 35.43	94.88%	0.999
Atp6 type A mtDNA	y=-3.34x+34.81	99.19%	0.999
Nad4 type B mtDNA	y=-3.26x+34.01	102.71%	0.999
Atp6 type B mtDNA	y=-3.31x+34.15	100.5%	0.999

Extended Data Table S2. Sequences of the qPCR primers

Gene	5'-Sequence-3'
N_ATPα_Frw	GATTTCGGTGGTGGATCTCTG
N_ATPα_Rev	GCTGGTCGAATACCCTTGAA
A_Nad4_Frw	ATTCCCTTTTCATTTGTGATTGCC
A_Nad4_Rev	GCAAACTATAAACAAACGTAGTTGACC
A_Atp6_Frw	CCCCGTTAATAGTGTTACTTGAGA
A_Atp6_Rev	CCCAGCTCAGCGATTAAG
B_Nad4_Frw	GTGTTAGTGAGGGTGTGTATGGG
B_Nad4_Rev	ATAGGGTCAGAAAAATTATTGCTGC
B_Atp6_Frw	TGTCTGTACCGTTAGGTTGCC
B_Atp6_Rev	TACCTGCCCACCAGCAAG

Extended Data Table S3. Conditions for qPCR reaction, PCR, LR-PCR

35 cycles qPCR		
Initial Denaturation (only once)	94°C	10 min
Denaturation	94°C	10s
Annealing (all primers)	60°C	30s
Extension	72°C	30s
**35 cycles PCR**		
Initial Denaturation (only once)	95°C	5 min
Denaturation	93.5°C	55s
Annealing (all primers)	*Tab.S4*	30s
Extension	72°C	80s
Final Extension	72°C	5min
**35 cycles LR-PCR**		
Initial Denaturation (only once)	98°C	30s
Denaturation	98°C	10s
Annealing (all primers)	*Tab.S4*	30s
Extension	72°C	8 min
Final Extension	72°C	10 min

Extended Data Table S4. Sequences of the PCR and Long Range PCR primers

Gene	5'-Sequence-3'	Temp.
BRS1M	GTGCTAGTAAGAGTGTGTGTGAGTGTGG	66℃
BRS2MR	AAGACCTAACATTAAGCTAGGAGCGACA	
BRS2M	ATCTGTGCCTCTAGGTTGCCCTTTAGGT	66℃
BRS1MR	CCACACTCACACACACTCTTACTAGCACTC	
BRS1F	CTTATTAGAGTGTTAGTGAGGGTGTGTATGGG	64℃
BRS2FR	TAACCACACCCATACACACCCTCACTAA	
BRS2F	ATGTCTGTACCGTTAGGTTGCCCTCTAG	64℃
BRS1FR	GCATTAAACCAGGGGCAACAAAGAAA	
BRS2A	CTCTTTAGGAATCTGCACTTTGTTCT	58℃
BRS3A	CCACCACCGCTAAACTCTACATTA	
BRS2B	TTCTTTAGGGATTTGTTCTTTGTTTC	58℃
BRS3B	CACCACCACTGAACTCTACGTTG	

Extended Data Table S5 mtDNA to nDNA ratio for A and B type mitochondrial genome

	mtA_Nd4/nDNA	mtA_Atp6/nDNA	mtB_Nd4/nDNA	mtB_Atp6/nDNA
01 F_Mantle	2931,8 ± 652,5	3004,4 ± 849,8	0,0 ± 0,0	0,0 ± 0,0
02 F_Mantle	142,9 ± 22,0	132,0 ± 13,6	735,6 ± 77,7	754,7 ± 96,2
03 M_Mantle	10,9 ± 1,7	11,5 ± 2,4	69,4 ± 13,6	67,5 ± 16,1
05 F_Mantle	191,5 ± 42,4	190,3 ± 27,7	557,2 ± 127,5	576,1 ± 100,5
06 F_Mantle	0,0 ± 0,0	0,0 ± 0,0	477,9 ± 76,8	499,6 ± 47,3
08 M_Mantle	27,4 ± 5,1	26,8 ± 4,2	0,0 ± 0,0	0,0 ± 0,0
09 M_Mantle	32,1 ± 5,4	34,0 ± 6,8	0,0 ± 0,0	0,0 ± 0,0
11 M_Mantle	19,6 ± 5,9	19,9 ± 5,6	1,0 ± 0,3	1,1 ± 0,4
14 M_Mantle	0,0 ± 0,0	0,0 ± 0,0	23,7 ± 6,0	24,5 ± 5,7
16 F_Mantle	394,6 ± 52,5	367,5 ± 27,4	143,6 ± 24,0	155,1 ± 14,4
20 M_Mantle	29,3 ± 3,6	30,9 ± 3,6	0,0 ± 0,0	0,0 ± 0,0
23 M_Mantle	0,0 ± 0,0	0,0 ± 0,0	22,6 ± 2,2	21,7 ± 2,8
27 F_Mantle	0,0 ± 0,0	0,0 ± 0,0	1107,5 ± 460,4	919,2 ± 274,5
28 M_Mantle	0,0 ± 0,0	0,0 ± 0,0	25,7 ± 3,2	26,6 ± 1,2
31 F_Mantle	0,0 ± 0,0	0,0 ± 0,0	756,5 ± 45,9	614,7 ± 50,2
35 M_Mantle	19,8 ± 2,1	23,7 ± 1,2	0,0 ± 0,0	0,0 ± 0,0
36 M_Mantle	0,0 ± 0,0	0,0 ± 0,0	275,2 ± 15,1	265,1 ± 13,1
40 F_Mantle	0,0 ± 0,0	0,0 ± 0,0	421,8 ± 39,2	443,1 ± 40,7
47 M_Mantle	0,0 ± 0,0	0,0 ± 0,0	27,3 ± 2,3	23,0 ± 5,1
48 M_Mantle	0,0 ± 0,0	0,0 ± 0,0	33,5 ± 3,7	37,2 ± 2,9
49 M_Mantle	0,0 ± 0,0	0,0 ± 0,0	22,4 ± 2,5	22,5 ± 2,4
53 F_Mantle	0,0 ± 0,0	0,0 ± 0,0	2387,6 ± 122,3	2731,7 ± 288,0
56 F_Mantle	287,7 ± 39,8	309,7 ± 74,2	0,0 ± 0,0	0,0 ± 0,0
58 M_Mantle	276,5 ± 48,0	274,9 ± 75,9	0,0 ± 0,0	0,0 ± 0,0
60 M_Mantle	54,1 ± 2,1	55,5 ± 1,3	19,3 ± 1,4	21,1 ± 1,1
66 F_Mantle	253,6 ± 25,5	233,2 ± 14,8	856,1 ± 71,5	871,9 ± 112,3
68 M_Mantle	0,0 ± 0,0	0,0 ± 0,0	37,7 ± 5,4	30,3 ± 3,4
70 F_Mantle	0,0 ± 0,0	0,0 ± 0,0	951,4 ± 107,4	1003,3 ± 94,5
71 F_Mantle	582,1 ± 62,4	610,4 ± 66,8	0,0 ± 0,0	0,0 ± 0,0
72 M_Mantle	0,0 ± 0,0	0,0 ± 0,0	34,6 ± 6,1	30,0 ± 5,1

Extended Data Table S6 qPCR results for nuclear gene *atpα*

ATPα nDNA	Cq	Cq SD	copies	SD
01 F_Mantle	28,29	0,17	115,6	13,3
02 F_Mantle	23,34	0,1	3270,4	230,8
03 M_Mantle	20,72	0,14	19169,0	1817,8
05 F_Mantle	24,49	0,09	1506,3	94,5
06 F_Mantle	22,14	0,04	7354,7	178,9
08 M_Mantle	19,66	0,15	39375,5	4121,5
09 M_Mantle	19,34	0,15	48847,7	4710,3
11 M_Mantle	18,9	0,2	66348,2	9067,3
14 M_Mantle	18,96	0,13	63364,6	5788,1
16 F_Mantle	22,24	0,06	6866,7	269,1
20 M_Mantle	18,44	0,09	89920,2	5356,1
23 M_Mantle	18,52	0,06	84582,5	3368,9
27 F_Mantle	23,36	0,17	3222,1	369,3
28 M_Mantle	18,74	0,02	73142,7	936,8
31 F_Mantle	24,3	0,05	1710,8	58,3
35 M_Mantle	20,5	0,05	22234,8	726,0
36 M_Mantle	25,07	0,04	1014,0	25,1
40 F_Mantle	24,42	0,09	1578,3	98,8
47 M_Mantle	18,14	0,11	110118,4	8257,3
48 M_Mantle	19,47	0,04	44667,7	1257,5
49 M_Mantle	18,28	0,1	100202,3	6541,8
53 F_Mantle	27,03	0,04	269,4	7,2
56 F_Mantle	23,49	0,14	2962,7	283,0
58 M_Mantle	24,04	0,13	2041,6	178,9
60 M_Mantle	23,65	0,02	2647,0	39,3
66 F_Mantle	26,04	0,02	525,9	5,5
68 M_Mantle	21,34	0,13	12657,6	1116,6
70 F_Mantle	24,97	0,08	1087,2	60,8
71 F_Mantle	25,25	0,06	896,6	33,9
72 M_Mantle	20,79	0,16	18386,7	1977,4
NTC	31,29	1,55	21,9	16,2

Extended Data Table S7 qPCR results for A-type mitochondrial gene *nad4*

Nad4 A mtDNA	Cq	Cq SD	copies	SD
01 F_Mantle	16,35	0,12	338810,3	27586,4
02 F_Mantle	15,87	0,11	467292,7	33849,2
03 M_Mantle	17,07	0,07	209814,9	10149,6
05 F_Mantle	16,6	0,22	288511,9	41704,4
06 F_Mantle	29,79	0,07	43,3	2,1
08 M_Mantle	14,62	0,09	1077164,0	65300,8
09 M_Mantle	14,05	0,08	1569631,0	85557,0
11 M_Mantle	14,34	0,19	1300839,0	157341,7
14 M_Mantle	-	-	no	amplif.
16 F_Mantle	13,24	0,13	2709635,0	240417,0
20 M_Mantle	13,28	0,08	2634880,0	147203,1
23 M_Mantle	-	-	no	amplif.
27 F_Mantle	-	-	no	amplif.
28 M_Mantle	30,3	0,08	30,7	1,7
31 F_Mantle	32,08	-	non spec.	amplif.
35 M_Mantle	15,96	0,11	440802,6	31523,2
36 M_Mantle	30,09	0,11	35,4	2,6
40 F_Mantle	-	-	no	amplif.
47 M_Mantle	30,5	-	non spec.	amplif.
48 M_Mantle	27,07	0,03	264,5	5,1
49 M_Mantle	-	-	no	amplif.
53 F_Mantle	-	-	no	amplif.
56 F_Mantle	14,97	0,04	852304,5	25286,4
58 M_Mantle	15,59	0,11	564439,6	39905,2
60 M_Mantle	17,64	0,04	143105,9	3377,9
66 F_Mantle	17,75	0,13	133378,7	11884,0
68 M_Mantle	31,95	0,27	non spec.	amplif.
70 F_Mantle	30,56	0,01	non spec.	amplif.
71 F_Mantle	15,7	0,1	521862,8	34120,4
72 M_Mantle	32,15	-	non spec.	amplif.
NTC	33,68	1,19	5,5	10,2

Extended Data Table S8 qPCR results for A-type mitochondrial gene *atp6*

Atp6 A mtDNA	Cq	Cq SD	copies	SD
01 F_Mantle	16,31	0,19	347208,6	46778,3
02 F_Mantle	15,98	0,04	431636,5	10852,7
03 M_Mantle	16,96	0,13	219985,1	20283,0
05 F_Mantle	16,58	0,11	286606,0	21055,5
06 F_Mantle	28,67	0,3	69,7	13,3
08 M_Mantle	14,69	0,05	1055465,0	36622,4
09 M_Mantle	14,03	0,12	1661527,0	141858,7
11 M_Mantle	14,37	0,16	1317293,0	138215,2
14 M_Mantle	30,47	0,06	non spec.	amplif.
16 F_Mantle	13,42	0,05	2523792,0	82205,0
20 M_Mantle	13,28	0,07	2776247,0	137625,5
23 M_Mantle	30,41	0,17	non spec.	amplif.
27 F_Mantle	30,09	0,41	non spec.	amplif.
28 M_Mantle	29,46	0,12	40,2	3,3
31 F_Mantle	31,23	0,15	non spec.	amplif.
35 M_Mantle	15,69	0,03	527969,7	9544,6
36 M_Mantle	29,29	0,24	45,3	7,2
40 F_Mantle	31,85	0,04	non spec.	amplif.
47 M_Mantle	29,93	0,18	29,0	3,6
48 M_Mantle	26,76	0,03	256,4	4,7
49 M_Mantle	30,68	0,15	non spec.	amplif.
53 F_Mantle	32,26	0,04	non spec.	amplif.
56 F_Mantle	14,89	0,17	917493,9	111108,7
58 M_Mantle	15,61	0,24	561251,2	92114,5
60 M_Mantle	17,55	0,01	146809,4	1088,3
66 F_Mantle	17,81	0,08	122648,9	6414,7
68 M_Mantle	31,09	0,26	non spec.	amplif.
70 F_Mantle	30,07	0,15	non spec.	amplif.
71 F_Mantle	15,64	0,1	547258,4	36969,4
72 M_Mantle	31,12	0,1	non spec.	amplif.
NTC	32,27	0,83	6,6	3,5

Extended Data Table S9 qPCR results for B-type mitochondrial gene *nad4*

Nad4 B mtDNA	Cq	Cq SD	copies	SD
01 F_Mantle	33,52	0,01	non spec.	amplif.
02 F_Mantle	13,21	0,04	2405577,0	66260,4
03 M_Mantle	14,05	0,11	1330575,0	110386,0
05 F_Mantle	14,85	0,2	839333,3	127289,1
06 F_Mantle	12,68	0,18	3514488,0	465885,3
08 M_Mantle	30,17	0,29	non spec.	amplif.
09 M_Mantle	32,21	0,12	non spec.	amplif.
11 M_Mantle	18,26	0,2	68821,2	9934,6
14 M_Mantle	13,89	0,19	1504174,0	207888,8
16 F_Mantle	14,48	0,17	985742,1	119842,4
20 M_Mantle	28,26	0,18	58,4	7,5
23 M_Mantle	13,54	0,08	1911973,0	99877,0
27 F_Mantle	12,8	0,32	3568526,1	904533,5
28 M_Mantle	13,57	0,15	1877137,0	205961,3
31 F_Mantle	14,09	0,03	1294114,0	31728,7
35 M_Mantle	28,09	0,22	73,2	12,3
36 M_Mantle	16,41	0,04	279010,1	7991,6
40 F_Mantle	15,03	0,03	665723,7	16252,6
47 M_Mantle	12,9	0,01	3011449,0	10684,7
48 M_Mantle	13,89	0,11	1497971,0	117134,9
49 M_Mantle	13,31	0,06	2241630,0	92242,0
53 F_Mantle	15,08	0,03	643159,9	14794,0
56 F_Mantle	-	-	no	amplif.
58 M_Mantle	29,84	0,2	non spec.	amplif.
60 M_Mantle	18,66	0,08	51149,4	2777,9
66 F_Mantle	15,59	0,1	450235,7	32495,0
68 M_Mantle	15,65	0,06	477139,0	20645,8
70 F_Mantle	14,41	0,07	1034334,0	52368,3
71 F_Mantle	27,49	0,15	100,2	10,9
72 M_Mantle	15,1	0,07	636210,9	31152,0
NTC	32,71	1,57	4,4	4,6

Extended Data Table S10 qPCR results for B-type mitochondrial gene *atp6*

Atp6 B mtDNA	Cq	Cq SD	copies	SD
01 F_Mantle	30,4	0,28	non spec.	amplif.
02 F_Mantle	13	0,07	2468288,0	118328,5
03 M_Mantle	13,9	0,17	1292990,0	157236,1
05 F_Mantle	14,5	0,14	867717,4	87480,1
06 F_Mantle	12,4	0,1	3674073,0	249770,7
08 M_Mantle	29,6	0,3	non spec.	amplif.
09 M_Mantle	30	0,41	non spec.	amplif.
11 M_Mantle	18,1	0,25	71154,7	12269,5
14 M_Mantle	13,7	0,17	1553502,0	188546,4
16 F_Mantle	14,2	0,07	1064986,0	53473,0
20 M_Mantle	28,6	0,03	47,2	0,9
23 M_Mantle	13,4	0,12	1837255,0	154250,8
27 F_Mantle	12,7	0,22	2961797,0	443616,3
28 M_Mantle	13,3	0,04	1943398,0	58183,7
31 F_Mantle	14,2	0,06	1051615,0	47137,8
35 M_Mantle	28,1	0,17	69,1	8,2
36 M_Mantle	16,2	0,03	268776,8	6283,9
40 F_Mantle	14,8	0,03	699312,1	16470,3
47 M_Mantle	13	0,18	2533541,0	327211,5
48 M_Mantle	13,6	0,07	1659890,0	78971,3
49 M_Mantle	13,1	0,05	2255770,0	77300,5
53 F_Mantle	14,7	0,11	735847,6	55739,3
56 F_Mantle	29,1	0,2	non spec.	amplif.
58 M_Mantle	29,3	0,2	non spec.	amplif.
60 M_Mantle	18,4	0,05	55861,1	2013,8
66 F_Mantle	15,4	0,17	458561,2	53638,0
68 M_Mantle	15,7	0,02	383694,1	5367,7
70 F_Mantle	14,2	0,05	1090811,0	35981,5
71 F_Mantle	27,4	0,04	107,8	3,1
72 M_Mantle	15,2	0,06	551919,3	23835,4
NTC	30	0,74	21,1	16,3

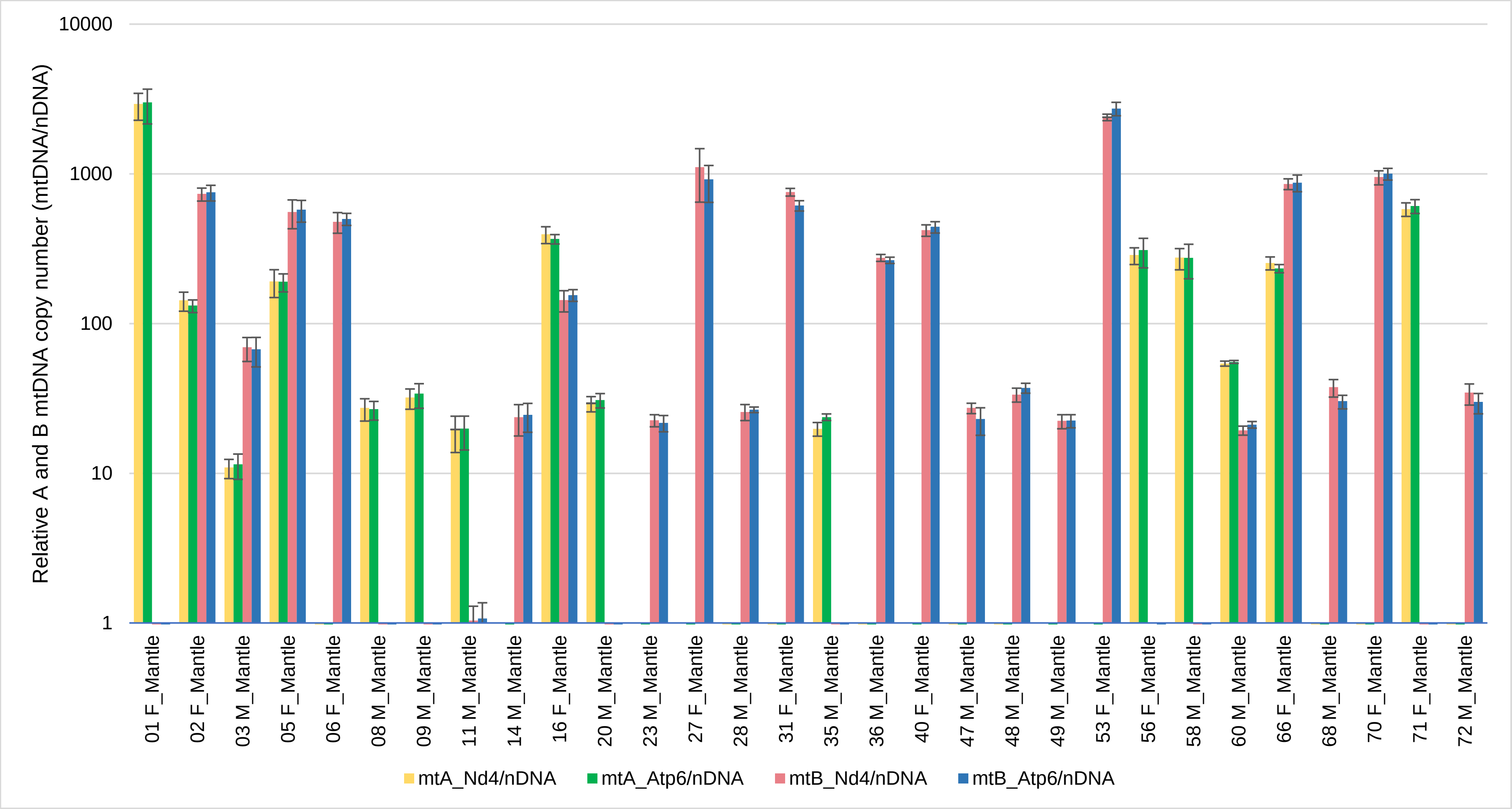

Extended Data Figure S1. Number of mitochondrial genomes (mtDNA type A and B; *Nad4* and *Atp6* genes for both mitogenomes) in relation to copies of nuclear DNA (atpα).

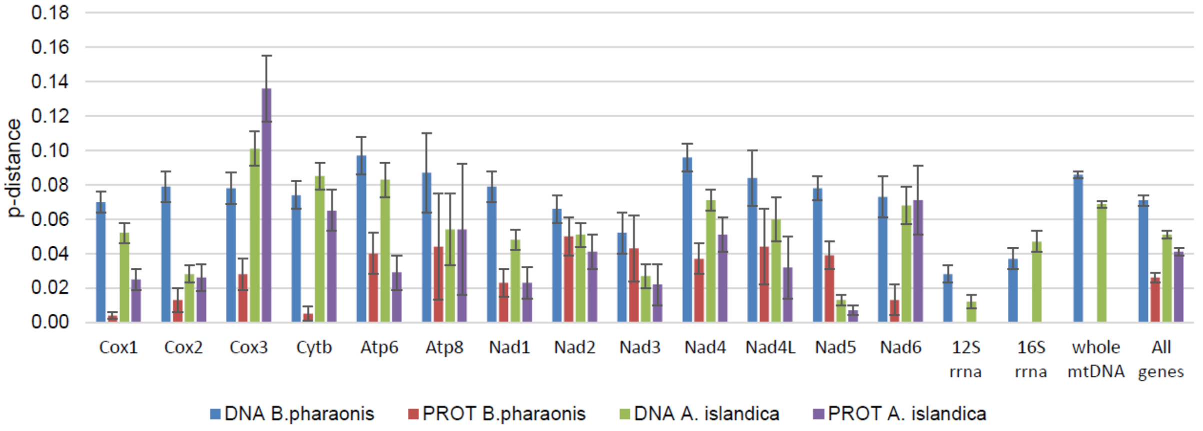

Extended Data Figure S2. Divergence between A and B type mitogenome of *B. pharaonis* and M and F type mitogenome of *Arctica islandica*. Blue and green color represent p-distance for nucleotide sequences, Dark red and violet color represent p-distance for translated protein gene
